# Twenty‐one Entrusted Professional Activities in German Dental Education—Day‐One Competencies

**DOI:** 10.1002/jdd.13931

**Published:** 2025-05-02

**Authors:** Nejra Kosić, Susanne Gerhardt‐Szép

**Affiliations:** ^1^ Department of Operative Dentistry Center of Dentistry and Oral Medicine (Carolinum) Goethe University Frankfurt Frankfurt am Main Germany

**Keywords:** clinical skills and assessment, dental education, entrustable professional activities (EPAs)

## Abstract

**Introduction:**

In medical education, entrusted professional activities (EPAs) have been an integral part of the curriculum, primarily being used in clinical traineeships and postgraduate medical education. The use of EPAs in postgraduate medical education is transferable to dental training, as clinical skills are already part of the curriculum. The data from this study could help propose dental EPAs that reflect competencies expected in the professional field, potentially aiding in more effectively evaluating student competencies and standardizing assessment criteria.

**Methods:**

After research on the use of EPAs in dental education across Europe, Asia, and the United States, 21 EPAs were chosen, covering all areas currently taught in the German dental graduate program, and matched with the domains of the National Catalog of Learning Objectives in Dentistry. Following, an electronic survey was developed and distributed to dentists working at university hospitals in the Rhine‐Main region, as well as in private practices. Participants were asked to evaluate each EPA statement on a 6‐point Likert scale and encouraged to provide qualitative feedback on the EPAs.

**Results:**

120 participants responded to the survey (20.7%). Overall, there was substantial agreement on the EPAs chosen to represent the tasks expected of young dentists on their first day in practice. Participants also provided qualitative feedback in the form of optional comments. Concerns raised included the perceived lack of experience for certain tasks.

**Conclusion:**

The data from this study suggest that the proposed EPAs accurately reflect the professional tasks expected of newly graduated dentists on their first day in practice.

## 1 Introduction

In medical education, entrusted professional activities (EPAs) have been an integral part of the curriculum for some time. Currently, the primary use of EPAs is in clinical traineeships and postgraduate medical education. The concept of EPAs was first introduced by the working group of Olle ten Cate as a practical framework for implementing competency‐based medical education [1, 2, 3]. He defines an EPA as “an essential task of a discipline that enables a person to perform their work without supervision once sufficient competence has been demonstrated” [4]. EPAs help turn competencies into observable actions in a practical setting, bridging the gap between theoretical knowledge and clinical practice [5, 6]. The use of EPAs in postgraduate medical education is applicable to dental training since clinical skills are already part of the curriculum. EPAs play a critical role in addressing gaps in dental education by providing a structured framework that connects theoretical knowledge to real‐world clinical skills. The shift from time‐based progression in traditional dental education to a competency‐based approach ensures that students master essential skills and demonstrate readiness to practice before advancing [7]. A notable gap in dental education is the limited hands‐on clinical exposure, especially in advanced procedures or complex patient communication. EPAs address this by defining specific tasks—such as performing a root canal treatment or managing difficult patient interactions—that students must demonstrate proficiency in before advancing. This ensures that students gain the necessary experience to practice competently and safely. However, the topic of EPAs is still a new concept in dental education, especially in Germany.

Currently, the National Catalog of Learning Objectives in Dentistry (Nationaler Kompetenzbasierter Lernzielkatalog Zahnmedizin [NKLZ]) [8] defines the skills dental graduates should have acquired by the time they obtain their license to practice in Germany. It aligns with the legal requirements of the Dental Licensing Regulations (Zahnärztliche Approbationsordnung [ZApprO]) [9]. Besides specifications on specialist knowledge, the NKLZ covers clinical skills, professional behavior, and communication competencies. It consists of 26 Chapters, listing almost 160 competencies, 268 sub‐competencies, 1785 learning objectives, 71 consultation occasions, and 131 diseases related to dentistry. It sets the foundation for dental education in Germany for curricular design, examinations, and teaching methods [10].

As part of the reform of the ZApprO [8], which came into force in 2019, students are now required to treat patients across all dental disciplines as part of new integrated courses. This opens the possibility of implementing EPAs in Germany, similar to the Student‐Run Dental Clinic at Radboud University Nijmegen [11]. One of the main reasons cited for introducing EPAs in dentistry is to help improve students' learning success and simplify the assessment of their clinical skills [12]. Studies show that EPAs are becoming increasingly important in dental education because, unlike medicine, dental education includes not only the development of clinical skills but also a specialized training component. In medicine, specialized training happens only after graduation. After obtaining their license, dentists must be entrusted to perform these skills independently, much like in veterinary education, where students must also practice relevant professional activities unsupervised by the time they enter professional practice [13, 14]. Therefore, the primary goal of dental education is to prepare students to transition successfully from the learning environment into professional practice. This study aims to survey experienced dentists in the Rhine‐Main region to find out which competencies are expected of dental graduates when they begin working independently after obtaining their license. The data from this study could help create a set of dental EPAs that better reflect the competencies expected in the professional field. These EPAs could help improve how student competencies are assessed and make the evaluation process more consistent.

## 2 Methods

The study was reviewed by the Ethics Committee of Goethe University Frankfurt am Main in March 2024, and it was determined that an ethics vote was not required.

### 2.1 EPA Development Process

**FIGURE 1:**
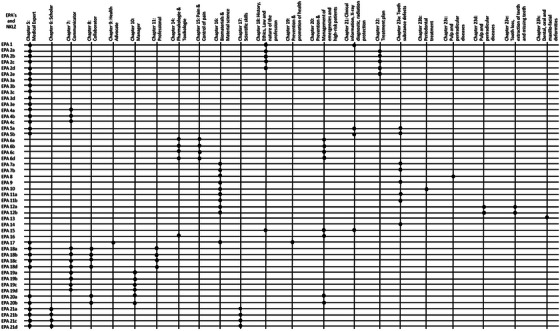
Entrustable Professional Activities (EPA) matched to corresponding National Catalog of Learning Objectives in Dentistry (NKLZ) domains. The black dots indicate a significant connection.

In the first phase, we conducted extensive research on the use of EPAs in dental education across Europe, Asia, and the United States to determine which EPAs were compatible with the requirements of the German dental curriculum. Ultimately, 21 EPAs were chosen, covering all areas currently taught in the dental graduate program, and the first version of the EPA form was developed (Appendix). The second phase involved an initial Delphi round (*n* = 2), where each EPA was matched with the domains of the NKLZ [7], the reference document that defines the learning objectives for dental education in Germany. This ensured that the EPAs chosen aligned with current requirements (Figure 1).

In the third phase, through another Delphi process (*n* = 30), a qualitative review of the EPAs was conducted to request feedback from general dentists, specialists, and educational researchers at the Center of Dentistry and Oral Medicine, Goethe University Frankfurt, to refine the wording, design, and breadth of the EPA statements. Feedback mostly focused on breaking down the EPAs into individual components, making it easier to assess each statement. The feedback was incorporated, and the final version of the 21 EPA statements was produced. The fourth phase consisted of developing an electronic survey, which was then distributed to participants.

### 2.2 Sample Size

The sample consisted of dentists working at university hospitals in the Rhine‐Main region (Frankfurt am Main, Gießen, Marburg, Mainz [population of greater area = 5.9 million]), as well as dentists in private practices participating in the dental clinical traineeship and career exploration program. Contact details for dentists at the university hospitals were acquired from the websites of the institutions and the internal register of the Center of Dentistry and Oral Medicine (Carolinum gGmbH) at the Goethe University Frankfurt. Contact details for private practitioners were obtained via the Hesse Dental Association portal and the website of the Clinic and Polyclinics for Dental, Oral, and Maxillofacial Diseases at Mainz University Medical Center. All participants who entered their e‐mail addresses on the aforementioned websites or whose e‐mail address was published via a practice website were contacted. Dentists who did not work at one of the university hospitals in the Rhine‐Main region, or who were not participating in the clinical traineeship or career exploration program were excluded.

A total of 536 dentists were ultimately contacted via email to distribute the survey between April 2 and 16, 2024. Participants were asked to evaluate each EPA statement on a 6‐point Likert scale, ranging from 1 (strongly disagree) to 6 (strongly agree). Participants were also encouraged to provide qualitative feedback on the EPAs, such as modifications, additions, or suggestions, and to indicate the university from which they graduated.

### 2.3 EPA Evaluation Process

In April 2024, an electronic survey was created and distributed via email using the SoSci Survey platform (SoSci Survey GmbH, Munich, Germany). The email included a cover letter explaining the background of the study, as well as details about the anonymous, voluntary nature of participation and an estimate of the time needed to complete the questionnaire. Contact information for further questions and a direct link to the survey were also provided. The survey was distributed in three rounds, with the first sent in April, requesting the completion of the questionnaire within 14 days, and reminders sent in May. Due to a low response rate, a third reminder was sent in early June, to all those who had not yet responded, asking for completion within seven days. The survey period ended on June 18, 2024.

### 2.4 Statistics

Data collection occurred through the survey platform, and statistical analysis was conducted using IBM SPSS Statistics software (Version 29.0.2.0; IBM Germany GmbH). This descriptive study recorded quantitative results using means, standard deviations, medians, minimum and maximum values, and nominal results using absolute and relative frequencies. Cronbach's alpha was used to assess the internal consistency of the Likert items, and Kruskal‐Wallis tests were applied to compare the perceived importance of skills across different universities. Qualitative feedback was analyzed using a coding framework. The responses were categorized into themes, which were summarized to suggest modifications to the EPAs.

## 3 Results

**FIGURE 2:**
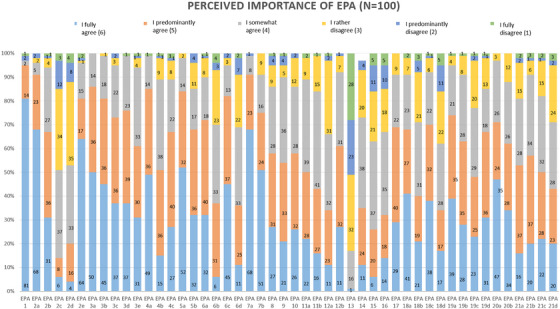
Surveyed perceptions of the importance of each Entrustable Professional Activities (EPA). The numbers on the bars represent the prevalence with which each option was selected. The total height of each bar reflects 100% of the responses.

A total of 536 dentists were contacted for the survey, representing all five divisions (operative dentistry, prosthodontics, oral surgery, periodontics, orthodontics) of dentistry in the German educational program. Of these, 120 participants responded (20.7%), but twenty responses were excluded in the analyses, due to incompleteness, resulting in 100 evaluable questionnaires (see Table 1). Overall, there was substantial agreement on the EPAs chosen to represent the tasks expected of young dentists on their first day in practice (Figure 2). EPAs with lower ratings included those related to treatment planning (EPA 2c), prognosis assessment (EPA 2d), and oral care for special‐needs patients (EPA 15). The lowest‐rated out of the 21 was the EPA related to orthodontic treatment (EPA 13).

**TABLE 1:** Surveyed ratings and evaluations of Entrustable Professional Activities (EPA) using a 6‐point Likert scale (*n* = 100).

EPA	I fully agree (6)	I predominantly agree (5)	I somewhat agree (4)	I rather disagree (3)	I predominantly disagree (2)	I fully disagree (1)	Mean
1 – Can obtain a patient's medical history	81%	14%	2%	—	2%	1%	5.69
2a—Can examine the patient	68%	23%	5%	2%	2%		5.53
2b—Can establish a diagnose	31%	36%	27%	4%	1%	1%	4.89
2c—Can establish a comprehensive treatment plan	6%	8%	37%	34%	12%	3%	3.53
2d—Can assess the prognosis	4%	16%	33%	35%	8%	4%	3.61
2e—Can obtain informed consent	64%	17%	15%	1%	1%	2%	5.36
3 – Has specialist knowledge about:							
3a—The etiology of the most common dental diseases	50%	36%	14%	—	—	—	5.36
3b—The pathogenesis of the most common dental diseases	45%	36%	18%	1%	—	—	5.25
3c—The clinical symptoms of the most common dental diseases	37%	36%	22%	3%	2%	—	5.03
3d—The diagnosis of the most common dental diseases	37%	39%	23%	—	—	1%	5.1
3e—The therapy of the most common dental diseases	31%	30%	33%	4%	1%	1%	4.83
4a—Can acquire the results of diagnostic and screening tests	49%	36%	14%	—	—	1%	5.31
4b—Can interpret the results of diagnostic and screening tests	15%	36%	38%	9%	1%	1%	4.52
4c—Can communicate the results of diagnostic and screening tests	27%	40%	22%	8%	1%	2%	4.78
5a—Understands the contribution that imaging and other diagnostic techniques can make in achieving a diagnosis	52%	32%	14%	2%	—	—	5.34
5b—Can use basic imaging equipment and carry out an examination as appropriate to the case, in accordance with good health and safety practice and current regulations	32%	36%	17%	11%	4%	—	4.81
6a—Can provide control of pain during the provision of care	32%	40%	18%	8%	1%	1%	4.91
6b—Can provide control of anxiety during the provision of care	6%	31%	33%	23%	3%	4%	4.02
6c—Can perform local anesthesia during the provision of care	45%	37%	13%	3%	2%	—	5.2
6d—Can provide behavioral techniques during the provision of care	11%	25%	33%	22%	7%	2%	4.05
7a—Can perform direct adhesive restorative treatments	68%	23%	8%	—	1%	—	5.57
7b—Can perform direct non‐adhesive restorative treatments	51%	24%	16%	9%	—	—	5.17
8 – Can perform endodontic treatment	27%	31%	28%	9%	4%	1%	4.65
9 – Can perform indirect restorative treatment	21%	33%	36%	5%	4%	1%	4.59
10 – Can provide periodontal treatment	26%	32%	28%	12%	2%	—	4.68
11a—Can provide fixed prosthetic treatment	22%	28%	39%	9%	—	2%	4.57
11b—Can provide removable prosthetic treatment	16%	27%	41%	15%	—	1%	4.41
12a—Can perform simple surgical care to establish, preserve, and restore oral health and function	11%	23%	32%	31%	2%	1%	4.07
12b—Can perform simple non‐surgical care to establish, preserve, and restore oral health and function	27%	34%	31%	7%	1%	—	4.79
13 – Can provide simple orthodontic treatment	1%	—	16%	32%	23%	28%	2.4
14 – Can manage simple acute dental trauma	11%	24%	38%	20%	4%		4.09
15 – Can provide oral care for special care groups (e.g. children, elderly patients)	6%	20%	37%	21%	11%	5%	3.74
16 – Can manage emergencies in the dental setting	14%	18%	35%	18%	10%	5%	3.93
17 – Can provide preventive, maintenance, and follow‐up care to promote health	29%	40%	22%	9%	—	—	4.89
18 – Can communicate effectively, using language appropriate to the audience concerned and in full respect of confidentiality and privacy:							
18a—With patients	41%	27%	23%	7%	—	2%	4.96
18b—With the public	21%	19%	31%	21%	5%	3%	4.21
18c—With professional colleagues	38%	32%	22%	6%	1%	1%	4.97
18d—With responsible authorities	17%	17%	28%	22%	11%	5%	3.92
19 – Can develop trusting relationships and sustained partnerships to deliver person‐centered care with the following groups:							
19a—Individuals	39%	35%	21%	4%	—	1%	5.06
19b—Families	28%	35%	28%	8%	—	1%	4.8
19c—Communities	23%	25%	29%	20%	2%	1%	4.44
19d—Other professionals	31%	36%	18%	13%	1%	1%	4.8
20a—Can collaborate as a member of an inter‐professional team	47%	24%	26%	3%	—	—	5.15
20b—Can provide care as a member of an inter‐professional team	34%	28%	26%	12%	—	—	4.84
21 – Has an understanding of and competence in the logical approaches to:							
21a—Scientific reasoning	16%	37%	28%	15%	2%	2%	4.44
21b—Clinical reasoning	20%	37%	34%	6%	1%	3%	4.63
21c—The distinction between a and b	22%	28%	32%	15%	1%	2%	4.49
21d—The strengths and limitations between a and b	20%	23%	28%	24%	2%	3%	4.26

The highest‐rated EPAs were related to obtaining the patient's history (EPA 1), examining the patient (EPA 2a), obtaining informed consent (EPA 2e), knowledge about the etiology of dental diseases (EPA 3a), acquiring diagnostic and screening results (EPA 4a), and performing adhesive and non‐adhesive restorative treatment (EPAs 7a and 7b). Practical skill‐related EPAs were not necessarily rated higher than those related to knowledge or communication. Participants also had the opportunity to provide qualitative feedback in the form of optional comments. Concerns raised mostly included the perceived lack of experience for certain tasks. For example, one participant shared that being able to perform complex endodontic treatment (EPA 8) is not expected of a newly licensed dentist, only the treatment of simple cases is expected, as the ability and the knowledge of how to approach cases that are more challenging comes with experience. One participant noted that communication with patients and colleagues (EPA 18) requires innate talent, which may not be fully learned in a formal educational setting. The survey showed that there was no significant (p‐value = 1) difference in the perceived importance of skills across universities and that experienced dentists hope to see in young colleagues starting their careers.

## 4 Discussion

This study describes the development and assessment of an initial version of EPAs describing baseline competencies expected of young dentists entering the professional practice. The evaluation was conducted by 100 dentists representing diverse practice areas, including educators and experienced practitioners. It shows an overall high level of agreement with the proposed EPAs. In the future, these EPAs could play a significant role in dental education, as they describe competencies and skills executed daily by dentists.

By implementing EPAs, dental schools could standardize student performance expectations, ensuring graduates meet consistent standards of practice regardless of their institution. This standardization could reduce variability in clinical competency and address gaps caused by differing teaching opinions and methods. As healthcare becomes more collaborative, EPAs encourage interprofessional education, promoting teamwork and communication between dental students and other healthcare providers. This aligns with the increasing emphasis on collaborative, patient‐centered care. Finally, EPAs are designed to reflect real‐world tasks that dentists perform in practice. This helps students transition more smoothly from the classroom to professional practice, filling the gap left by traditional academic assessments, which may not fully capture the complexities of daily clinical scenarios. By focusing on these key areas, EPAs address gaps in dental education, ensuring that graduates are well‐prepared to meet the challenges of modern dental practice.

The EPA development process emerged after the implementation of the novel Dental Licensing Regulations [9]. It was based on a comparison of various methods in the literature with each having its advantages and disadvantages and no optimal strategy mentioned [4, 11, 13, 15–20]. This was followed by a systematic review of international studies and existing frameworks in the medical, dental, and veterinary fields [11, 17, 21–23]. After selecting the EPAs, they were aligned with the NKLZ to ensure they reflected the competencies necessary for dental practice in Germany (Figure 1).

The EPA framework could be further refined by using a more deliberate process. This would include multiple rounds of feedback and collaboration, leading to greater commitment and diversity of thought [24]. Future improvements at a more practical level could be achieved by, for example, applying the proposed template for writing dental EPAs by Tonni et al [25]. This correlates to the proposed EPA template described for medical EPAs [4]. Therefore, the next steps might include clearer descriptions of each EPA and developing standardized assessment methods. Faculty training would also be needed to ensure consistent evaluation [26].

The findings of this study should be taken into account along with its limitations. Firstly, the response rate of 20.7% is on the lower end. The lower the response rate in a survey, the higher the chance of sampling bias. However, surveys among healthcare providers often show a low and even declining response rate [27, 28, 29]. Therefore, our response rate of 20.7% can be classified as average. Due to the low response rate, our findings cannot be generalized and therefore cannot be considered representative of all dentists.

Secondly, the EPA framework created in this study is specific to one institution. It was comprised of examples from medicine, pharmacy, veterinary medicine, and dentistry across the United States, Asia, and Europe, which were adapted to German dental education. However, individuals from different institutions in Germany may find different EPAs more important, based on different teaching opinions and their perceived needs. Thirdly, the study is limited to one region in Germany and reflects the views of only a small sample. Although private practice dentists are affected the most by the skills that newly qualified dentists bring, as they are the ones offering them the opportunity to work independently for the first time, they are also the most difficult group to attract and engage in surveys. On one hand, this could be due to the demanding nature of daily practice, where the focus is on treating patients. On the other hand, private practice dentists are less involved in teaching activities. As a result, they may not be as interested in this topic, as university‐based dentists and educators, who are involved in teaching and curriculum development on a daily basis. The length of the questionnaire should also be considered. With 21 EPAs, our questionnaire can be considered quite long compared to other studies in dental fields, where the maximum was 15 EPAs. Shortening the questionnaire could have a positive impact on increasing the response rate and therefore improve representativeness. The study also used a survey to evaluate the importance of the EPAs. In other health professions, QUEPA has been used to collect similar data [30]. Additionally, alternative methods like the EQual instrument could provide more substantial feedback as a result [31].

It is an interesting subject, how these EPAs will be evaluated in the future. Dental education in Germany is currently undergoing a major change with the implementation of the new ZApprO [9]. For example, the EPA regarding orthodontic treatment (EPA 13) received the lowest rating. This is due to the fact that until now, the focus of orthodontics in dental education has been on theoretical knowledge, dental technology skills, and case planning, rather than clinical skills. With the newly integrated courses, orthodontic treatment is an essential component of education early on. This means that future dental graduates will enter the professional field with a more refined competency portfolio than current graduates. This could result in a shift in the expectations that experienced professionals hold regarding newly licensed colleagues.

In other health professions, EPAs have proven to be a useful tool for assessing and measuring learners’ improvements and capabilities. Therefore, it is likely that EPAs will fulfill the same expectations in dentistry. EPAs could further advance dental education programs across Germany by outlining concrete tasks that are exclusive to each division and equalizing assessment across faculty.

## 5 Conclusion

The data from this study suggest that the presented EPAs accurately reflect the professional tasks expected of newly graduated dentists on their first day of practice. Given the limited number of studies on EPAs in dental education, especially in German‐speaking countries, this analysis may encourage further research. It could eventually lead to the development and implementation of additional EPAs in dentistry.

## Conflicts of Interest

The authors declare no conflicts of interest.
